# A Picture Is Worth a Thousand Views: A Triple Crossover Trial of Visual Abstracts to Examine Their Impact on Research Dissemination

**DOI:** 10.2196/22327

**Published:** 2020-12-04

**Authors:** Sandra Oska, Edgar Lerma, Joel Topf

**Affiliations:** 1 William Beaumont School of Medicine Oakland University Rochester, MI United States; 2 College of Medicine University of Illinois at Chicago Chicago, IL United States; 3 Associates in Nephrology, SC Chicago, IL United States

**Keywords:** social media, science communication, visual abstract, Twitter, dissemination

## Abstract

**Background:**

A visual abstract is a graphic summary of a research article’s question, methods, and major findings. Although they have a number of uses, visual abstracts are chiefly used to promote research articles on social media.

**Objective:**

This study aimed to determine if the use of visual abstracts increases the visibility of nephrology research shared on Twitter.

**Methods:**

A prospective case-control crossover study was conducted using 40 research articles published in the *American Journal of Nephrology* (AJN). Each article was shared by the AJN Twitter account in 3 formats: (1) the article citation, (2) the citation with a key figure from the article, and (3) the citation with a visual abstract. Tweets were spaced 2 weeks apart to allow washout of the previous tweet, and the order of the tweets was randomized. Dissemination was measured via retweets, views, number of link clicks, and Altmetric scores.

**Results:**

Tweets that contained a visual abstract had more than twice as many views as citation-only tweets (1351, SD 1053 vs 639, SD 343) and nearly twice as many views as key figure tweets (1351, SD 1053 vs 732, SD 464). Visual abstract tweets had 5 times the engagements of citation-only tweets and more than 3.5 times the engagements of key figure tweets. Visual abstract tweets were also associated with greater increases in Altmetric scores as compared to citation-only tweets (2.20 vs 1.05).

**Conclusions:**

The use of visual abstracts increased visibility of research articles on Twitter, resulting in a greater number of views, engagements, and retweets. Visual abstracts were also associated with increased Altmetric scores as compared to citation-only tweets. These findings support the broader use of visual abstracts in the scientific community. Journals should consider visual abstracts as valuable tools for research dissemination.

## Introduction

**Figure 1:**
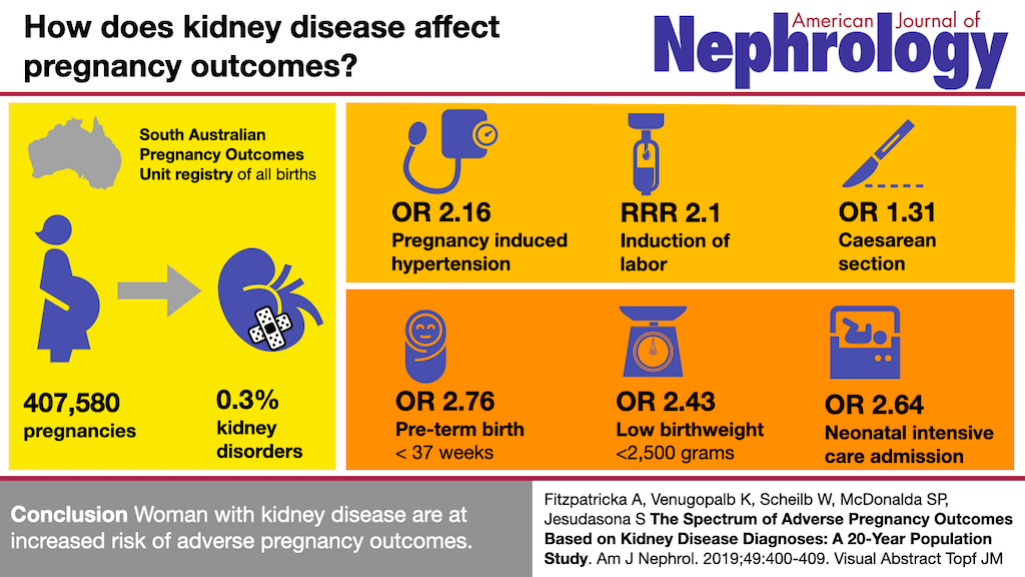
A sample visual abstract published alongside an American Journal of Nephrology original research publication.

The visual abstract is a graphic summary of a research article’s question, methods, and major findings (Figure 1) [1]. Although visual abstracts were initially conceived as a way to visually represent studies for slide presentations, they were quickly adapted to promote and share studies on social media. Similar to a text abstract, a visual abstract does not replace the act of reading an article, but instead allows a viewer to quickly grasp what question a study addresses, how the investigators examined this question, and the associated results. Visual abstracts are used to accompany scientific articles on various social media platforms that rely heavily on image-based communication. Twitter, specifically, is frequently used to share research findings and generate conversations among medical professionals and scientists [2-6]. Twitter allows physicians, journals, and institutions to disseminate research beyond the traditional readership of scientific publications to a broader audience comprised of researchers, clinicians, and the general public.

The utility and impact of the visual abstract was first evaluated by Ibrahim et al [7]. The *Annals of Surgery* shared 44 original research articles via Twitter twice each, once with only the article title and hyperlink, and once with the article title, hyperlink, and visual abstract. Ibrahim et al found that tweets with the visual abstract had a 7-fold increase in impressions (views), an 8-fold increase in retweets, and a nearly 3-fold increase in article visits [7]. Although the authors concluded that visual abstracts were responsible for the increased dissemination, it is possible that the inclusion of *any image* would have produced similar results. Our study evaluates whether visual abstracts improve viewership more than the inclusion of relevant key figures. Additionally, the visual abstract study by Ibrahim et al [7] was conducted when the use of visual abstracts was still novel. It is possible that as the use of visual abstracts has increased on social media, the associated effects have attenuated. In this study, we aim to assess the impact of visual abstract tweets as compared to key figure and citation-only tweets.

## Methods

### Overview

Between December 2018 and October 2019, a prospective, crossover study was conducted using original research articles published in the *American Journal of Nephrology* (AJN). Every article that had a visual abstract during the study period was included in the study. Three were excluded from analysis due to technical difficulties with the tweets or the AJN website. All research articles published in the sections “Patient-Oriented, Translational Research,” “Laboratory Investigation,” and “Transplantation” had visual abstracts. “Novel Research Findings” was the only section in which published research articles did not have visual abstracts. Editorials, review articles, errata, and letters did not have visual abstracts and were not included.

Each article was tweeted from the AJN Twitter account (*@AmJNephrol*) in 3 formats:

1. Citation-only
  tweets included the study title, the authors, and author institutions. If the authors or institutions had a presence on Twitter, links to those accounts were also included. All tweets had links to the original article in AJN.
2. Key figure
  tweets included the citation-only information along with a key figure from the article. The figure was chosen by one of the investigators (SO).
3. Visual abstract
  tweets included the citation-only information along with a visual abstract created by the AJN visual abstract team.

**Figure 2:**
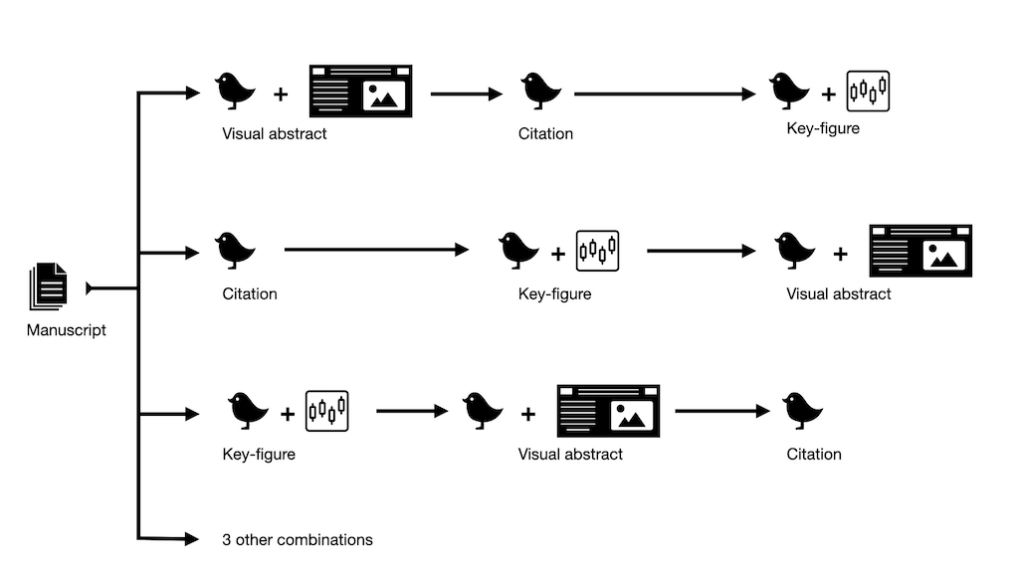
Schematic representation of how each article was tweeted 3 times.

To minimize confounding from the order of tweets, each article was randomized into 1 of 6 groups. Randomization was done using the random number generator in Microsoft Excel to assign every manuscript to one of 6 groups. Each group represented the order in which the visual abstract tweet, key figure tweet, and citation-only tweet would be posted. For example, articles randomized to group 1 were tweeted in the order of visual abstract, followed by citation-only, followed by key figure (Figure 2). The rest of the groups were a permutation of this order. For each article, a 2-week “washout” period was employed between tweets. Additionally, the number of followers that the AJN account had at the time of each tweet was recorded.

### Assessment and Data Extraction

All analysis was performed using SAS 9.4 (SAS Institute Inc). Descriptive statistics were obtained for the outcome variables. All outcome variables were analyzed using negative binomial models. Negative binomial models were used because the data are count data, as all possible values of the variables were positive and the variables were positive integers. The engagement rate was analyzed using One-Way Analysis of Variance (ANOVA).

Altmetric scores were recorded for each study before each tweet and at the end of each washout period. Altmetric tracks the attention that research outputs receive online [8]. Altmetric scores are calculated using an automated algorithm that weighs the amount of attention a scholarly work has received in social and traditional media. The absolute difference in Altmetric scores was calculated for all tweets. Twitter analytics [9] was used to measure impressions (views), total engagements, retweets, article visits, and engagement rates for tweets. Twitter analytics defines engagements as the total number of users that interacted with a tweet. Interactions include retweets, replies, likes, following the Twitter account, clicking on a link or hashtag, playing embedded media, clicking on the author’s profile photo or name, or expanding the tweet. Link clicks represent the number of times users clicked the link in the tweet taking users to the study on the AJN website. The engagement rate is the number of engagements divided by the number of views.

## Results

The study included 40 original nephrology publications. The 40 publications were randomized to Groups 1-6: Group 5 contained 5 publications (12.5%); Groups 1-4 and Group 6 had 7 publications each (17.5%). In citation-only tweets, the average number of impressions, engagements, retweets, and link clicks did not differ significantly by group (*P*=.58, *P*=.23, *P*=.77, and *P*=.32, respectively). In key figure tweets, there was no significant difference by group for average impressions, engagements, retweets, and link clicks (*P*=.07, *P*=.29, *P*=.58, and *P*=.57, respectively). For visual abstract tweets, the average number of impressions, engagements, retweets, and link clicks did not significantly differ by group (*P*=.08, *P*=.20, *P*=.64, and *P*=.37, respectively).

Visual abstract tweets had more than twice as many views as citation-only tweets (1351, SD 1053 vs 639, SD 343; *P*<.001) and nearly twice as many views as key figure tweets (1351, SD 1053 vs 732, SD 464; *P*<.001) (Table 1). Visual abstracts had 5 times the engagement of citation-only tweets (*P*<.001) and more than 3.5 times the engagement of key figure tweets (*P*<.001). Visual abstract tweets had a higher engagement rate (5.7%) than both citation-only tweets (2.4%; *P*<.001) and key figure tweets (3.2%; *P*<.001).

There was no significant difference in the number of link clicks for visual abstract tweets and key figure tweets (*P*=.35), or for visual abstract tweets and citation-only tweets (*P*=.35). The increase in Altmetric scores was significantly higher (*P*=.02) for the 2 weeks following a visual abstract tweet than for the 2 weeks following a citation-only tweet. There was no significant difference in Altmetric score for the 2 weeks following a key figure tweet compared to the 2 weeks following a visual abstract tweet (*P*=.85).

**Table 1:** Comparison of measures between tweet types.

	Citation-only tweet	Key figure tweet	VA^a^ tweet	*P* value for VA vs citation only	*P* value for VA vs key figure
Number of impressions	638.58	731.88	1351.08	<.001	<.001
Number of total engagements	15.75	24.08	83.75	<.001	<.001
Number of retweets	0.95	1.30	3.68	<.001	<.001
Number of link clicks	8.13	6.68	8.95	.35	.35
Difference in Altmetric scores	1.05	2.33	2.20	.02	.85
Engagement rate	3.18	2.46	5.75	<.001	<.001

^a^Visual abstract.

## Discussion

### Overview

Visual abstracts increase the visibility of scientific publications on Twitter. In our crossover trial, the inclusion of a visual abstract roughly doubled the number of Twitter accounts that saw a tweet compared to a tweet without any image or compared to a tweet with a key figure from the article. However, views are passive. In order to assess how visual abstracts encourage more active forms of viewership, we tracked tweet engagement levels. Here, visual abstract tweets were even more impactful than both citation-only and key figure tweets, with visual abstracts increasing Twitter engagement by 5 fold and 3.5 fold, respectively. Although others have documented the effect of a visual abstract on research dissemination, this trial is, to our knowledge, the first to control for the effect of an image in the tweet. This is important because images in general increase views and engagement [10]. In order for visual abstracts to be a meaningful part of a dissemination strategy, they have to be more effective than a key figure from the article. The number of link clicks, however, did not differ significantly for visual abstract, key figure, and citation-only tweets. Although the online visibility of the articles improved with the addition of a visual abstract, the addition of a visual abstract did not cause users to click the link revealing the full article text. In the Ibrahim et al [7] crossover study, tweeting a visual abstract increased article visits by nearly three-fold; however, this still represented less than 1% of the total impressions. In fact, our link click rate of 0.6% with a visual abstract is similar to the 0.8% rate recorded by Ibrahim et al [7]. These very low link click rates solidify the message that although social media can boost article exposure, that exposure is shallow. Social media can convey a message quickly, but it rarely pulls viewers in for deeper consideration. Additionally, many viewers may be more interested in quickly viewing and evaluating the key elements of a paper rather than clicking through to the article link, which is often hidden behind a paywall.

A number of studies have assessed the effect of Twitter promotion on research impact and dissemination. In 2011, Eysenbach [11] showed that Twitter promotion was associated with future citations. A number of retrospective trials have reproduced these results [12,13]. A pair of subsequent randomized controlled trials from the same group were not able to show increased 30-day readership for articles randomized to twitter promotion [14,15]. However, in a trial of 696 cardiovascular articles randomized to twitter promotion or not, the tweeted group had a 1.4-fold increase in citations as well as increased Altmetric scores [16]. This was replicated by the Thoracic Surgery Social Media Network, which randomized 112 original scientific research articles from The Annals of Thoracic Surgery and The Journal of Thoracic and Cardiovascular Surgery to a tweeted group and a control group [17]. The articles were tweeted by one user and then retweeted by a team of 11 additional contributors (total audience of 52,893 followers). After 1 year of follow-up, the tweeted articles had over 4 times the number of citations as the nontweeted control group (3.1, SD 2.4 vs 0.7, SD 1.3). Of interest to our study, the authors found a high correlation with Altmetric score and citation count (R=0.72).

Several studies have demonstrated a positive association between Altmetric scores and citation rates. Azer and Azer [18] demonstrated that the Altmetric score for highly cited articles in medical professionalism literature published during or after 2007 was significantly correlated with citation counts. Similarly, Haddon Mullins et al [19] assessed the utility of Altmetric scores in general surgery literature and demonstrated that Altmetric scores had a significant positive correlation with citation number. Barbic et al [20] identified the 200 most frequently cited articles in the top 10 emergency medicine journals and concluded there was a low correlation between citation counts and Altmetric scores. The findings of such studies suggest that Altmetric scores can complement other tools and strategies that quantify research dissemination. A prospective trial by Luc et al [21] demonstrated that tweeting studies resulted in significantly more article citations over time and showed that the Altmetric score was an independent predictor of citations. In another prospective study, 24 articles were randomized to infographic or control groups and were disseminated through Twitter and Facebook [22]. Altmetric scores and abstract views were both higher in the infographic group than in the control group.

Only a few studies have looked at visual abstracts and their ability to promote articles [23]. In the previously mentioned Ibrahim et al [7] study, including a visual abstract resulted in a 7-fold increase in impressions, an 8-fold increase in retweets, and a nearly 3-fold increase in article visits. Thoma et al [24] assessed how various social strategies, including the use of infographics and podcasts, were able to promote articles from the *Canadian Journal of Emergency Medicine*. Although the study was not randomized, the authors found that when articles were promoted on social media using infographics, abstract views more than doubled and Altmetric scores nearly tripled . In a follow-up randomized controlled trial, the same group found that when articles were tweeted with infographics, abstract views and article reads doubled compared to abstract-only tweets [22]. Lindquist et al [25] demonstrated an over 6-fold increase in views in their N-of-1 experience of using a visual abstract for a geriatric medicine article.

Many journals are adopting social media strategies to promote the articles they publish [26]. However, simple strategies such as just tweeting the names of and links to articles have failed to improve readership or dissemination in multiple randomized controlled trials [15,27]. As social media becomes noisier, more sophisticated strategies are needed to rise above the din. Visual abstracts are part of this strategy and have been adopted by 95 journals. It takes humans 6 seconds to read 20 words, but the meaning of a visual symbol can be established in a quarter of a second [1]. This means that people casually scrolling through a social media feed can quickly glean the meaning of visual abstract, while a longer, text-based description may be ignored. Additionally, visual abstracts are turning up outside of social media feeds. Visual abstracts are regularly found in lectures and presentations at regional and national meetings, further promoting the article and the publishing journal [23].

### Principal Results

To our knowledge, this is the first study to demonstrate that the presence of a visual abstract uniquely boosts views and improves engagement. This demonstrates that the use of a visual abstract is more valuable than a relevant image from the article. The crossover nature of our study design limited the effect of multiple graphic designers with variable talent levels in creating visual abstracts. Additionally, each article was randomized to 1 of 6 groups. Each group corresponded to the order in which the visual abstract, figure, and text would be tweeted. This randomization was employed to minimize the effect of order on impressions, total engagements, engagement rate, retweets, link clicks, and Altmetric scores. As mentioned in the results, there was no significant difference between impressions, total engagements, engagement rate, retweets, or link clicks by group. Although the AJN twitter account gained 560 followers during the time of the study, the number of followers at the time of tweeting did not differ significantly by tweet type. The triple crossover design employed in our study allows articles to be compared to themselves and removes concerns that more authors, higher budgets, or home institutions with more engagement in promoting their faculty could alter the results.

### Limitations

This study does have a number of limitations. We only used Twitter analytics and Altmetric to assess dissemination of articles. As such, we cannot comment on whether the journal site was visited more frequently or whether the use of visual abstracts was associated with article downloads, an important finding in the landmark study by Ibrahim et al [7]. Because all studies were tweeted with and without a visual abstract, our study cannot determine if visual abstracts increase subsequent citations. Two of the authors (JT, EL) have large social media footprints, and their tweeting may have swayed the results. Ideally, their use of Twitter in regard to these articles should have been protocolized (either to tweet all of them or none of them). Although this was discussed during the study design, no such protocol was implemented. Additionally, we did not evaluate the tweeting patterns of the authors of the included articles. If authors were active on Twitter and had large social media footprints, this may have affected the magnitude of the results. However, by using a crossover design where each article serves as its own control, we feel that we have neutralized the effect of authors and institutions on Twitter. In addition, editorials, review articles, and letters did not have visual abstracts and were therefore not included in the study. The visual abstract design is less appropriate for such article types. Our study cannot comment on the utility of visual abstracts for these publication types.

### Comparison With Prior Work

While our study has some similarities to the original Ibrahim et al [7] study on visual abstracts, we found a smaller magnitude of effect. Possible explanations could be that as visual abstracts become more common, the effect size is smaller. Another possible explanation is that not all visual abstracts are created equal, and that the visual abstracts used in the landmark study [7] were better at attracting attention than the visual abstracts created for this study. Additionally, because both of these studies are single journal studies, the nature of the journal could be an unaddressed confounder. *The Annals of Surgery* is the official journal of the American Surgical Association, while AJN is a journal not associated with a professional society.

### Conclusions

This study demonstrates that the addition of a visual abstract increases the attention that an article attracts on social media. This further establishes the visual abstract as an effective tool to improve research dissemination on social media. Although a visual abstract is not a substitute for reading an article, the adoption of visual abstracts by journals can enhance the online visibility of original research. The effect size and significance of our findings support the adoption of visual abstracts by journals hoping to improve online visibility of recently published original research.

## Abbreviations

AJN: American Journal of Nephrology
